# Investigating hormesis, aging, and neurodegeneration: From bench to clinics

**DOI:** 10.1515/med-2024-0986

**Published:** 2024-06-17

**Authors:** Vittorio Calabrese, Uwe Wenzel, Tommaso Piccoli, Ursula M. Jacob, Lidia Nicolosi, Giovanni Fazzolari, Gabriella Failla, Tilman Fritsch, Naomi Osakabe, Edward J. Calabrese

**Affiliations:** Department of Biomedical and Biotechnological Sciences, University of Catania, Catania, Italy; Institut für Ernährungswissenschaft, Justus Liebig Universitat Giessen, Germany; Neurology Unit, Department of Biomedicine, Neuroscience and Advanced Diagnostics (BIND), University of Palermo, Palermo, Italy; System Biologie AG, Wollerau, Switzerland; NAM Institute, Salzburg, Austria; Department of Bio-Science and Engineering, Faculty of System Science and Engineering, Shibaura Institute of Technology, Tokyo, Japan; Department of Environmental Health Sciences, Morrill I, N344, University of Massachusetts, Amherst, MA 01003, United States of America; Department of Biomedical and Biotechnological Sciences, University of Catania, Catania, Italy

**Keywords:** antioxidants, hormesis, Nrf2, mitochondrial medicine, *C. elegans*, longevity

## Abstract

Mitochondria-derived reactive oxygen species production at a moderate physiological level plays a fundamental role in the anti-aging signaling, due to their action as redox-active sensors for the maintenance of optimal mitochondrial balance between intracellular energy status and hormetic nutrients. Iron regulatory protein dysregulation, systematically increased iron levels, mitochondrial dysfunction, and the consequent oxidative stress are recognized to underlie the pathogenesis of multiple neurodegenerative diseases, such as Parkinson’s disease and Alzheimer’s disease. Central to their pathogenesis, Nrf2 signaling dysfunction occurs with disruption of metabolic homeostasis. We highlight the potential therapeutic importance of nutritional polyphenols as substantive regulators of the Nrf2 pathway. Here, we discuss the common mechanisms targeting the Nrf2/vitagene pathway, as novel therapeutic strategies to minimize consequences of oxidative stress and neuroinflammation, generally associated to cognitive dysfunction, and demonstrate its key neuroprotective and anti-neuroinflammatory properties, summarizing pharmacotherapeutic aspects relevant to brain pathophysiology.

## 1 Introduction

Reduction of cellular expression and activity of antioxidant proteins and the resulting increase of oxidative stress are fundamental causes in the aging processes and neurodegenerative diseases [1]. There have been numerous theories to explain the aging process. Although several lines of evidence suggest that accumulation of oxidative molecular damage is a primary causal factor in senescence, it is increasingly evident that the mitochondrial genome may play a key role in aging and neurodegenerative diseases. Mitochondrial dysfunction is characteristic of several neurodegenerative disorders, and evidence for mitochondria being a site of damage in neurodegenerative disorders is partially based on decreases in respiratory chain complex activities in Parkinson’s disease (PD), Alzheimer’s disease (AD), and Huntington’s disease [2]. Such defects in respiratory complex activities, possibly associated with oxidant/antioxidant balance perturbation, are thought to underlie defects in energy metabolism and induce cellular degeneration. Mitochondria play also a key role in the anti-aging signaling, as moderate reactive oxygen species (ROS) production operates as redox sensors of intracellular nutrients and energy status to maintain optimal mitochondrial balance, although further understanding of mitochondrial supercomplex dynamics and functional energy metabolism, as well as physiological ROS generation and their regulation by nutrition and caloric intake are yet to be fully elucidated.

**Figure 1:**
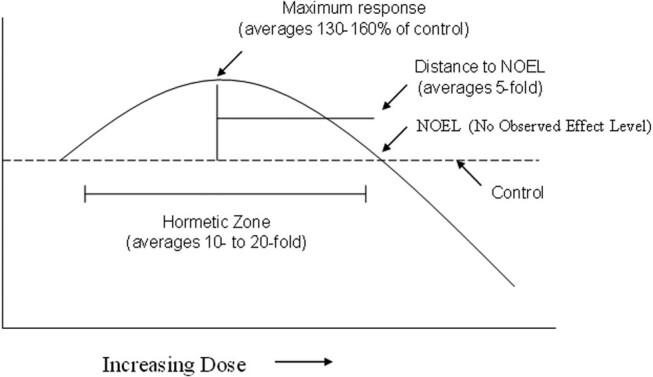
Dose–response curve depicting the quantitative features of hormesis.

During the last few years, cellular oxidant/antioxidant balance has become the subject of intense study, particularly by those interested in brain aging and in neurodegenerative mechanisms [3]. Several lines of evidence suggest that accumulation of oxidative molecular damage is a causal factor in senescence. The direct evidence for this hypothesis is that overexpression of antioxidative genes for Cu, Zn-superoxide dismutase and catalase in transgenic *Drosophila melanogaster* prolongs the life span, retarding the age-associated accumulation of oxidative damage [3]. Among the correlative evidence supporting the involvement of oxidative stress are the following: (1) oxidative molecular damage to DNA and proteins increases exponentially with age, and concomitantly, the rates of mitochondrial \[{\text{O}}_{\text{2}}^{\cdot -}]\] and H_2_O_2_ generation as well as the susceptibility of tissues to experimentally induced oxidative stress are increased; (2) experimental regimens that extend life span, such as caloric restriction in mammals and reduction of metabolic rate in insect, decrease the accumulation rates of oxidative damage; (3) mitochondria make rather contradictory contributions to cell survival, as they are crucial for cellular energy metabolism, produce at significant rate oxygen and nitrogen free radicals, but also undergoing dynamic changes, through fusion and fission to adapt to different cellular states, modulate cellular neural cell signaling, brain aging, and neuronal cell death. An emerging interest is now focusing on exogenous small molecules that are capable of activating these systems as a novel target to minimize deleterious consequences associated with free radical-induced cell damage, such as during neurodegeneration or in cancer [4]. Hormesis has been conceptually adopted in biology and medicine to encompass adaptive responses of cells and organisms to moderate stress and, as such, will foster future research in applied neurosciences [3,4]. When mild stress occurs, this induces the activation of signaling pathways which are associated with the setting of a programmed cell life survival and withstanding to more severe stress conditions. The concept of hormesis has emerged as a significant dose–response model in toxicology and pharmacology [5]. The enhanced recognition of hormesis has occurred principally because traditional dose–response models such as the threshold model have not been able to account for the occurrence of nonrandom biological activity below well-established thresholds of response [6]. The hormetic dose–response challenges long-standing beliefs about the nature of the dose–response in a low-dose zone, having the potential to affect significantly the design of pre-clinical studies and clinical trials as well as strategies for optimal patient dosing in the treatment of numerous diseases. The hormetic dose–response may be reliably described as stimulation in the low-dose zone, followed by an inhibitory response at higher doses [6]. The magnitude of the stimulatory response at maximum is typically modest, being only about 30–60% above that of the control response (Figure 1). The vast majority of stimulatory responses are less than twice the control value [7]. This is the most distinguishing characteristic of the hormetic dose–response, being its most consistent and reliable feature.

## 2 Mitochondria and ROS in aging and neurodegenerative disorders

Harman first proposed that mitochondria may have a central role in the process of aging [8]. According to this theory, free radicals generated through mitochondrial metabolism can act as a causative factor of abnormal function and cell death. Mitochondria are the cell’s most significant source of oxidants and *in vitro s*tudies have indicated that approximately 1–2% of electron flow through the ETC results in the univalent generation of superoxide. Moreover, various toxins in the environment can injure mitochondrial enzymes, leading to increased generation of free radicals that over the life span would eventually play a major role in aging [4]. Mitochondria are evolutionary organelles regulating brain energy metabolism and are central to neuronal signaling pathways, brain aging, and apoptotic cell death [3]. These organelles undergo continuous morphological and structural remodeling, pertaining to mitochondrial dynamics, where fusion and fission processes are essential events to assure proper functioning in different redox states of cells [9]. The classically recognized mitochondrial function is the synthesis of ATP for energizing endergonic reactions; the other is the generation of ROS which may compromise the long-term survival of cells and constitute a major underlying cause of the aging process. Indeed, these two rather conflicting functions are part of the same process, namely mitochondrial respiration. More than 95% of the O_2_ taken up by the human body is used by mitochondrial cytochrome oxidase which adds four electrons to oxygen to generate a molecule of water. Cytochrome oxidase normally does not release ROS into its surroundings. However, a number of investigations have indicated that brain mitochondria undergo oxidative stress damage and a decrease in cytochrome *c* oxidase activity during aging [3]. Increasing evidence sustains the hypothesis that mitochondrial energy metabolism underlies the pathogenesis of neurodegenerative diseases. Decreased complex I activity is reported in the substantia nigra of post-mortem samples obtained from patients with PD [10]. Similarly, impaired complex IV activity has been demonstrated in AD [11]. Increased free radical-induced oxidative stress has been associated with the development of these disorders, and a large body of evidence suggests that NO˙ plays a central role. Loss of nigral glutathione is considered an early and crucial event in the pathogenesis of PD [12], and as a consequence, decreased peroxynitrite scavenging may also occur. Therefore, such perturbations in thiol homeostasis may constitute the starting point for a vicious cycle leading to excessive ONOO− generation in PD. In support of this, it has been reported that the selective inhibition of nNOS prevents 1-methyl-4-phenyl-1,2,3,6-tetrahydropyridine-induced Parkinsonism in experimental animals [13].

## 3 Nrf2 signaling and the vitagene network

**Figure 2:**
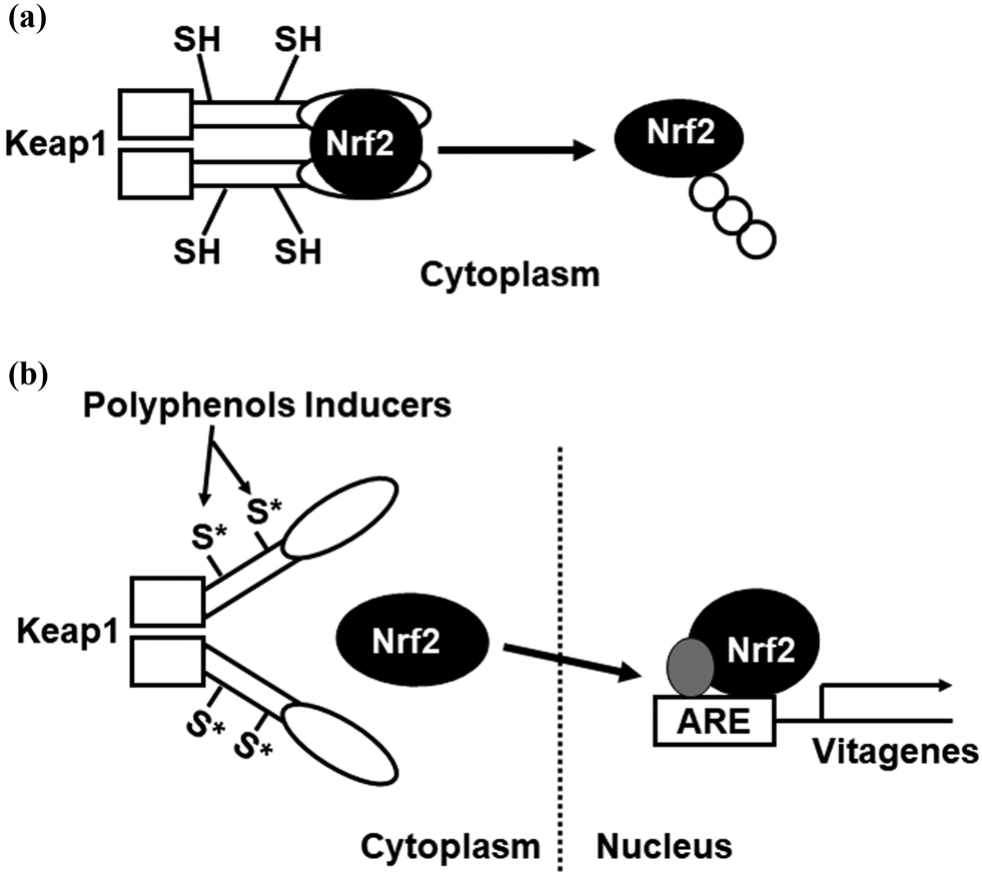
The Keap1/Nrf2/ARE pathway. (a) Under basal conditions, Nrf2 is bound to Keap1, a cytoplasmic repressor that targets Nrf2 for ubiquitination and proteasomal degradation. (b) Polyphenols, as molecules inducers, react with cysteine residues of Keap1, which operate as sensors, and hence induce stabilization of Nrf2, via entering into nucleus, where this transcriptional factor, by binding to the ARE, promotes transcriptional activation of cytoprotective vitagenes.

Among the cellular pathways conferring protection against oxidative stress, a key role is played by the products of vitagenes [1,14,15]. These include members of the heat shock protein (Hsp) family, such as heme oxygenase-1 and Hsp72, sirtuins, and the thioredoxin/thioredoxin reductase system [4,14,15]. The cellular stress response is regulated at the transcriptional, translational, and post-translational levels by a family of heat shock transcription factors (HSFs) that are expressed and maintained in an inactive state under non-stress conditions. HSFs, essential for all organisms to survive to acute or chronic stress, are also important for normal development and lifespan-enhancing pathways, and the repertoire of HSF targets has thus expanded well beyond the heat shock genes. Post-translational regulation of HSFs is emerging to integrate the metabolic state of the cell with stress biology, whereby controlling fundamental aspects of the health of the proteome and aging [4,15]. In addition to this, the KEAP1/Nrf2/ARE pathway is the basis of the cellular defense. Induction of this pathway has been shown to be protective against various stress conditions. On the other side, under conditions of Nrf2 deficiency with failure to upregulate this pathway, increased sensitization and accelerated disease pathogenesis have been demonstrated. Transcription factor Nrf2, under basal conditions, is continuously targeted for ubiquitination and proteasomal degradation by KEAP1, a protein acting as a repressor. It is well defined now that many inducers of the Nrf2 pathway chemically modify specific cysteine residues within KEAP1, leading to the loss of its ability to target Nrf2 for degradation. Subsequently, Nrf2 levels accumulate, enter the nucleus, bind as a heterodimer with a small Maf transcription factor to antioxidant response elements (AREs, specific sequences that are present in the promoter regions of NRF2-target genes), and hence activate transcription of NRF2-dependent vitagenes which encode a large network of cytoprotective proteins, including those that are involved in the metabolism and transport of a wide array of endo- and xenobiotics, proteins that have antioxidant functions, as well as those that participate in the synthesis, utilization, and regeneration of glutathione and NAD(P). Several phytochemicals act through the activation of transcription factor Nrf2 (Figure 2). Under basal conditions, these protective systems do not operate at maximum capacity but can be induced to higher activity levels by redox-active compounds, such as hormetic nutrients, thus reducing the risks of developing malignancies and multiple chronic diseases.

## 4 Nutritional polyphenols and their therapeutic potential

The intake of nutritional polyphenols has been shown to reduce the risk of vascular disease and enhance hippocampus-dependent cognitive function [16]. Continuous consumption of these foods can reduce the risk of chronic diseases and promote a healthy lifestyle [17]. Additionally, studies have found that the beneficial effects on the circulatory system, metabolic system, and brain function can persist for several hours after ingestion [18]. The mechanism by which nutritional polyphenols exert their biological activity is unknown due to their extremely low bioavailability [18]. However, recent studies suggest that their sensory properties, such as bitterness and astringency, act as mild stressors and maintain homeostasis [19]. Recently, it has been seen that mushrooms containing polyphenols, olive oil polyphenols, sulforaphane, or saffron counteract oxidative stress, mitochondrial dysfunction, and neuroinflammation closely connected to the initiation and progression of various brain disease conditions. Interestingly, these compounds exert antioxidant or toxic effects depending on their endogenous concentration and act according to the emerging principles of hormetic nutrition [20]. Following the hormetic paradigm, natural polyphenolic substances at low doses act as antioxidants in a wide range of brain diseases, this occurring through upregulation of Nrf2 signaling pathway and increased expression of vitagenes, such as NAD(P)H-quinone oxidoreductase (NQO1), glutathione transferase (GT), GPx, heme oxygenase-1 (HO-1), sirtuin-1 (Sirt1), and thioredoxin (Trx) system. This network, by blocking ROS production, plays a fundamental role in the free radical species metabolism, as well as in the biotransformation of xenobiotics, ultimately preventing neuronal cell death associated with neurotoxic damage. Importantly, dysregulation of the Nrf2 pathway in the central nervous system can be a prominent cause of selective neuronal susceptibility under oxidative and neuroinflammatory conditions, due to the high vulnerability of specific neuronal systems to oxidant/antioxidant balance perturbation. The neurobiological importance of mechanisms targeting Nrf2/vitagene pathways, relevant to the development of new therapeutical strategies capable of suppressing oxidative stress and neuroinflammation and the consequent cognitive dysfunction, occurring in all major neurodegenerative conditions, is here highlighted, underscoring innovative pharmacological perspectives based on neuronutrition in the hormetic low dose relevant to brain disorder management [19,21,22].

## 5 *Caenorhabditis elegans* as a model organism

In the nematode *C. elegans* such hormetic mechanisms can be perfectly studied based on its simplicity and genetic tractability. Models for AD and PD have been constructed through promoter, thus cell-specific transgenic expression of human genes encoding the amyloidogenic proteins β-amyloid or α-synuclein, respectively [23]. Transgenes encoding the pathogenic proteins in fusion with a fluorescent marker allow the degeneration of affected cells in the transparent organism by fluorescence microscopy. In a *C. elegans* strain expressing β-amyloid in body wall muscle cells, for instance, an olive-derived extract containing 20% of the polyphenol hydroxytyrosol reduces the rate of amyloid-β aggregates and also the β-amyloid-induced paralysis [24]. Those pathogenicity-reducing activities of hydroxytyrosol were diminished by the knockdown of two genes typically involved in hormetic responses, *skn-1/nrf-2* and *hsp-16.2* [25]. Similar results were obtained for the caloric restriction mimetic resveratrol from red grapes, where the inhibition of unfolded protein response and of proteasomal degradation and autophagy has been shown to prevent the activities of the polyphenol [16]. The polyphenol phlorizin from apple skins in dependence on Skn-1/Nrf-2 triggers autophagy and prevents β-amyloid-induced toxicity in an AD model but also the degeneration of dopaminergic neurons in a PD model of *C. elegans* [17]. Induction of autophagy was also proven as crucial for the neuroprotective effects of chlorogenic acid, an ester of caffeic acid and quinic acid, and a major phenolic compound in coffee [18]. Chlorogenic acid thereby reduced α-synuclein aggregation, improved motor disorders, and decreased ROS in a PD *C. elegans* model [18].

## 6 Conclusions

A large body of experimental evidence suggests that a functional interplay between ROS levels generated by mitochondria and various antioxidant pathways exert modulating effects on lifespan, ensuring brain-healthy aging and this phenomenon is hormesis based [26,27,28]. There is widespread evidence that the hormetic activation profile for ROS generation is in the 15–35% range greater than control values. Increases of ROS in the 200–250% range are associated with toxicity, that is, the upper end of the hormetic dose–response. The ROS-mediated hormetic stimulation is general, being reported for a wide range of chemical (e.g., caffeic acid, copper ions) and physical agents (i.e., non-ionizing and ionizing radiation), all conforming to this modest ROS increase for their stimulatory features than conform to the quantitative characteristic of the hormetic dose–response [29]. This striking commonality led Duan et al. [30] to state that “low concentrations of ROS from all sources exert a positive effect on cell proliferation.” Redox-active mechanisms based on oxidant/antioxidant balance, through cellular redox signaling, are subject of increasing interest in mitochondrial medicine, as a perturbation of this equilibrium is at the origin of several oxidant disorders, including chronic neurodegenerative diseases where ROS balance disruption is associated with mitochondrial dysfunction [1]. Consistent with this notion, based on the application of innovative medical and scientific technologies, anti-aging medicine emerges as a new science, conceptually defined as a medical specialty aimed at the early detection, prevention, treatment, and reversal of age-related disorders. It is a model of productive healthcare where innovation and research are conjugated to model new approaches capable of prolonging the healthy life span of humans. In this century, due to neurodegenerative diseases, cancer, and metabolic and heart diseases, assuring a healthy quality of life during aging is a crucial matter, as age-related pathologies have a substantially increasing impact on healthcare systems worldwide. It is well recognized to date how slowing of aging and age-related disorders can abolish more than 50% of all pathologies [2]. This can be achieved with strategies focusing on preventive and personalized medicine, delaying or reversing major age-associated neurodegenerative disorders. Translational application of these anti-aging and mitochondrial medicine concepts should allow efficient prevention, at early stages, of any of these diseases, where integration of detection, therapeutic interventions, and reversal of aging-related pathologies produce enhancement in the quality and in the extension of human lifespan, thus providing, at the same time, a new model for proactive healthcare policies for all this new millennium. Hormetic mechanisms are central to the possibility of targeted therapeutics in a pathway-specific manner at cell, tissue, and organismic levels and are appropriate intervention points in the AD and PD disease processes [31,32]. We propose and refine the potential of the therapeutic use of hormetic neuronutrients, such as natural polyphenols, in particular those highly active as Nrf2 inducers, primarily acting as Keap1-Nrf2 protein–protein interaction inhibitors, yet promoting Keap-1 degradation to regulate activation of Nrf2 related pathway. In this biochemical context, nutritional polyphenols are considered neuronutrients endowed with high potential as pharmacological modulators of neuroinflammation processes, thus providing a solid rationale for treating neurodegenerative disorders.
