# Corrigendum to “Investigating hormesis, aging, and neurodegeneration: From bench to clinics”

**DOI:** 10.1515/med-2024-2000

**Published:** 2024-12-19

**Authors:** Vittorio Calabrese, Uwe Wenzel, Tommaso Piccoli, Ursula M. Jacob, Lidia Nicolosi, Giovanni Fazzolari, Gabriella Failla, Tilman Fritsch, Naomi Osakabe, Edward J. Calabrese

**Affiliations:** Department of Biomedical and Biotechnological Sciences, University of Catania, Catania, Italy; Institut für Ernährungswissenschaft, Justus Liebig Universitat Giessen, Germany; Neurology Unit, Department of Biomedicine, Neuroscience and Advanced Diagnostics (BIND), University of Palermo, Palermo, Italy; Healthcare AG, Zurich, Switzerland; NAM Institute, Salzburg, Austria; Department of Bio-Science and Engineering, Faculty of System Science and Engineering, Shibaura Institute of Technology, Tokyo, Japan; Department of Environmental Health Sciences, Morrill I, N344, University of Massachusetts, Amherst, MA 01003, United States of America

In the published article Calabrese V, Wenzel U, Piccoli T, Jacob UM, Nicolosi L, Fazzolari G, Failla G, Fritsch T, Osakabe N, Calabrese EJ. Investigating hormesis, aging, and neurodegeneration: From bench to clinics. Open Med. (Wars) 2024; 19(1): 20240986, doi: 10.1515/med-2024-0986. PMID: 38911254 PMCID: PMC11193355, the affiliation of Ursula M Jacob is not correct.

The correct author affiliation is as follows:

Ursula M. Jacob: Healthcare AG, Zurich, Switzerland

